# Factors affecting satisfaction in patients with a rotator cuff retear: CT arthrography-based study

**DOI:** 10.1186/s12891-023-06617-1

**Published:** 2023-06-13

**Authors:** Bong Gun Lee, Joo-Hak Kim, Chang-Hun Lee, Seong Hyuk Eim, Kyeong-Jin Han, Wan-Sun Choi

**Affiliations:** 1 Department of Orthopaedic Surgery, Hanyang University College of Medicine, Seoul, Republic of Korea; 2 Department of Orthopaedic Surgery, Myoungji Hospital, Goyang, Republic of Korea; 3 Department of Orthopaedic Surgery, Ajou University School of Medicine, 164, World Cup-Ro, Yeongtong-Gu, Suwon-Si, Gyeonggi-Do Republic of Korea 16499

**Keywords:** Rotator cuff repair, Retear, Computed tomography arthrography, Patient satisfaction

## Abstract

**Purpose:**

The relationship between retear that may occur after rotator cuff repair and patient satisfaction is not well established. This study aimed to determine whether the types and size of the retear evaluated by computed tomography arthrography (CTA) influenced patient satisfaction. We also analyzed the patient factors that could affect patient satisfaction.

**Patients and methods:**

A total of 50 patients who were diagnosed with rotator cuff retear after undergoing arthroscopic rotator cuff repair were included in this study. All the patients were dichotomously classified into the satisfactory or dissatisfactory groups according to the patients’ self-classifications. CTA was used to assess the attachment status of the footprint, detect retear on the medial side of the footprint of the repaired cuff, and determine the retear size. Demographic factors, including sex, age, occupation, dominant upper extremity, duration of pain, presence of diabetes mellitus, trauma history, history of ipsilateral shoulder surgery, repair technique, worker’s compensation status, and functional shoulder score, were investigated.

**Results:**

Thirty-nine patients were classified into the satisfactory group and 11 patients were classified into the dissatisfactory group. There were no differences in age, sex, occupation, dominant hand, duration of pain, presence of diabetes mellitus, trauma history, history of ipsilateral shoulder surgery, repair technique, worker’s compensation, and duration of follow-up between the two groups. However, the postoperative American Shoulder and Elbow Surgeon (ASES) score (*P* < 0.01), visual analog scale (VAS) pain level (*P* < 0.01), anteroposterior (AP) length (*P* < 0.01), and area of the retear site (*P* < 0.01) were significantly different.

**Conclusion:**

The AP length and area of the retear site estimated using CTA were confirmed as the significant risk factors for dissatisfaction. However, the type of repaired rotator cuff judged by the attachment status of the footprint did not correlate with patient satisfaction. In addition, the postoperative VAS pain scale and ASES score was correlated with patient satisfaction.

## Introduction

Overall, the incidence of retear after rotator cuff repair has been observed to be approximately one in four patients [1]. However, numerous studies have reported that the clinical outcomes after rotator cuff repair improved regardless of whether retear had occurred. Therefore, they concluded that there was no correlation between rotator cuff repair integrity and the clinical outcomes [1–6]. These studies appear to validate rotator cuff repair as post-operative outcomes are good anyway; however, they may also raise fundamental questions about the necessity of rotator cuff repair owing to the irrelevance between repair integrity and clinical outcomes. On this issue, Tashjian said that despite the surgical fascination with healing, most studies have failed to show that anatomic healing makes an important difference with regard to the outcomes [7]. On the other hand, Yang et al. reported poor clinical outcomes in the retear group compared to the intact group in a systematic review of rotator cuff repair after retear, [8] and Kim et al. reported poor clinical outcomes in the retear group in a study of arthroscopic revision rotator cuff repair [9]. Thus, the relationship between repair integrity and clinical outcome has remained controversial until recently.

Some authors advocate that the above-mentioned “knowledge gap” occurs because non-anatomical factors could affect the clinical outcomes [10, 11]. However, we consider that the inaccuracy of evaluation methods for cuff integrity can also cause a knowledge gap. Several modalities, such as ultrasonography, magnetic resonance imaging (MRI)/MR arthrography (MRA), and CTA have been used to evaluate the postoperative cuff integrity in previous studies. Ultrasonography has a relatively low interobserver reliability; additionally, MRI/MRA can be used restrictively because of its high cost. In contrast, CTA cannot differentiate fatty infiltrates in rotator cuffs, but can be performed at a lower cost than MR, and the newly developed multi-detector CT (MDCT) has been reported to have a sensitivity of 99% and a specificity of 100% for the diagnosis of supraspinatus (SSP) tears [12].

Despite the high frequency of retears, overall patient satisfaction with rotator cuff repair is high. Many patients do not want to undergo revision surgery because they are not experiencing any discomfort despite the presence of an actual retear. The authors wanted to investigate the factors that may affect this unpredictable patient satisfaction by using CTA, a more intuitive and accurate imaging test. Thus, the aim of the present study was to determine whether the size and type of the retorn rotator cuff evaluated by CTA after arthroscopic rotator cuff repair influenced patient satisfaction. We also analyzed demographic factors that could affect patient satisfaction.

## Materials and methods

The study was approved by the local institutional review board of Hanyang University Hospital.

### Patients and demographic factors

A total of 50 patients diagnosed with rotator cuff retear after undergoing arthroscopic rotator cuff repair by a single surgeon (LBG) at a single institution between April 2014 and February 2019 were included in this retrospective study. A total of 423 patients underwent rotator cuff repair during this period. Of these, 401 patients underwent imaging at 6 months postoperatively and 362 underwent CTA, excluding 39 patients who underwent ultrasound or MRI. Finally, 50 patients had a retear detected on CTA. The patients included 22 men and 28 women, and their mean age at surgery was 62.6 years (range: 43–74 years).

Patients’ factors including sex, age, occupation, dominant upper extremity, duration of pain, presence of diabetes mellitus, preoperative rotator cuff tear size, trauma history, history of ipsilateral shoulder surgery, repair technique, worker’s compensation status, and duration of follow-up were investigated by chart review and a survey. The occupations were classified as labor-intensive or non-labor-intensive according to the patients’ self-judgment.

### Satisfaction and clinical outcomes evaluation

At the last follow-up, all the patients were classified into the satisfaction or dissatisfaction group in a dichotomous manner according to the patients’ self-classifications. The ASES score and VAS of pain level were recorded preoperatively and at the last follow-up. The preoperative and postoperative ranges of motion (ROMs), including forward flexion and external rotation of the involved shoulders, were assessed using a goniometer. The effects of the above-mentioned demographic factors, ASES score, VAS pain level, and shoulder ROM on patient satisfaction were analyzed.

### Computed tomography arthrography evaluation

All the patients underwent CTA 6 months after surgery. A MDCT (Somatom Definition, Siemens Medical Solutions, Erlangen, Germany) was used, and images were reconstructed with a scan thickness of 2 mm. CTA was performed with the patient supine after contrast injection into the glenohumeral joint by a radiologist under fluoroscopic guidance, and it was used to assess the attachment status of the footprint, detect retear on the medial side of the footprint of the repaired cuff, and determine the retear size. Since retear was regarded as a structural failure of the repaired rotator cuff, it was defined as a case in which the continuity of the rotator cuff was disrupted by the contrast agent, regardless of the biological healing status of the repaired area. There was some lack of coverage for massive tears involving the infrasupinatus and subscapularis, but supraspinatus tears were completely repaired in all patients. The evaluation of rotator cuff retears primarily focused on the supraspinatus because it was the area that can be accurately evaluated in the coronal plane of a CTA.

**Fig. 1:**
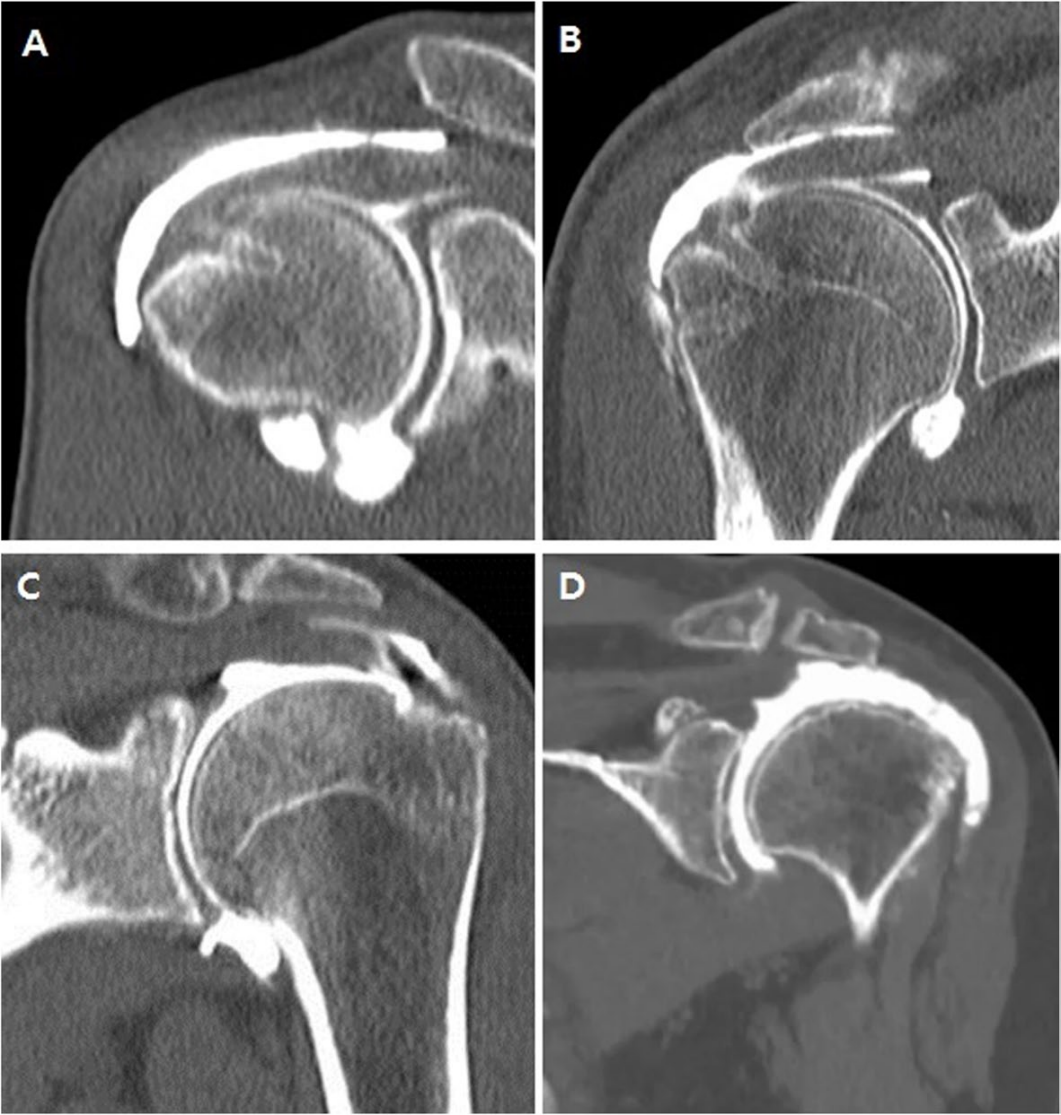
According to our CT arthrography repaired rotator cuff classification system, (**A**) is type Ia (footprint: full attachment, medial integrity: intact), (**B**) is type Ib (footprint: full attachment, medial integrity: dye leakage), (**C**) is type IIb (footprint: partial attachment, medial integrity: dye leakage) and (**D**) is type III (footprint retear)

In our experience, the pattern of retear after cuff repair can be divided into two cases: failure of the footprint to heal or new tears occurring proximal to the footprint. These two cases were combined to classify the postoperative CTA patterns. The postoperative CTA types I, II, III were classified depending on the healing status of the cuff footprint. Subtypes a and b were classified by the presence or absence of a tear on the medial side of the cuff. Full attachment of the rotator cuff on the footprint area on CTA was regarded as complete healing of the cuff footprint and classified as type I. Partial attachment on the footprint area print was regarded as incomplete healing and classified as type II. Type III was defined as a full detachment of the rotator cuff on the footprint area, which indicated footprint retear of the rotator cuff. Subtype “a” defined if there was no leakage of dye in the medial side of footprint of rotator cuff. When the dye leakage was shown in the medial side of footprint on one or more sections of CTA, i.e., more than 2 mm wide, it was defined as subtype “b” (Table 1, Fig. 1). According to these definitions, type Ia and IIa were excluded from the retear group.

**Table 1:** The types of repaired rotator cuff by CT arthrography

Type	Subtype	Footprint attachment	Medial tissue integrity	Status of repaired cuff
I	a	Full attachment	Intact	Healing
	b	Full attachment	Dye leakage in one or more images	Retear
II	a	Partial attachment	Intact	Healing
	b	Partial attachment	Dye leakage in one or more images	Retear
III		Footprint retear		Retear

**Fig. 2:**
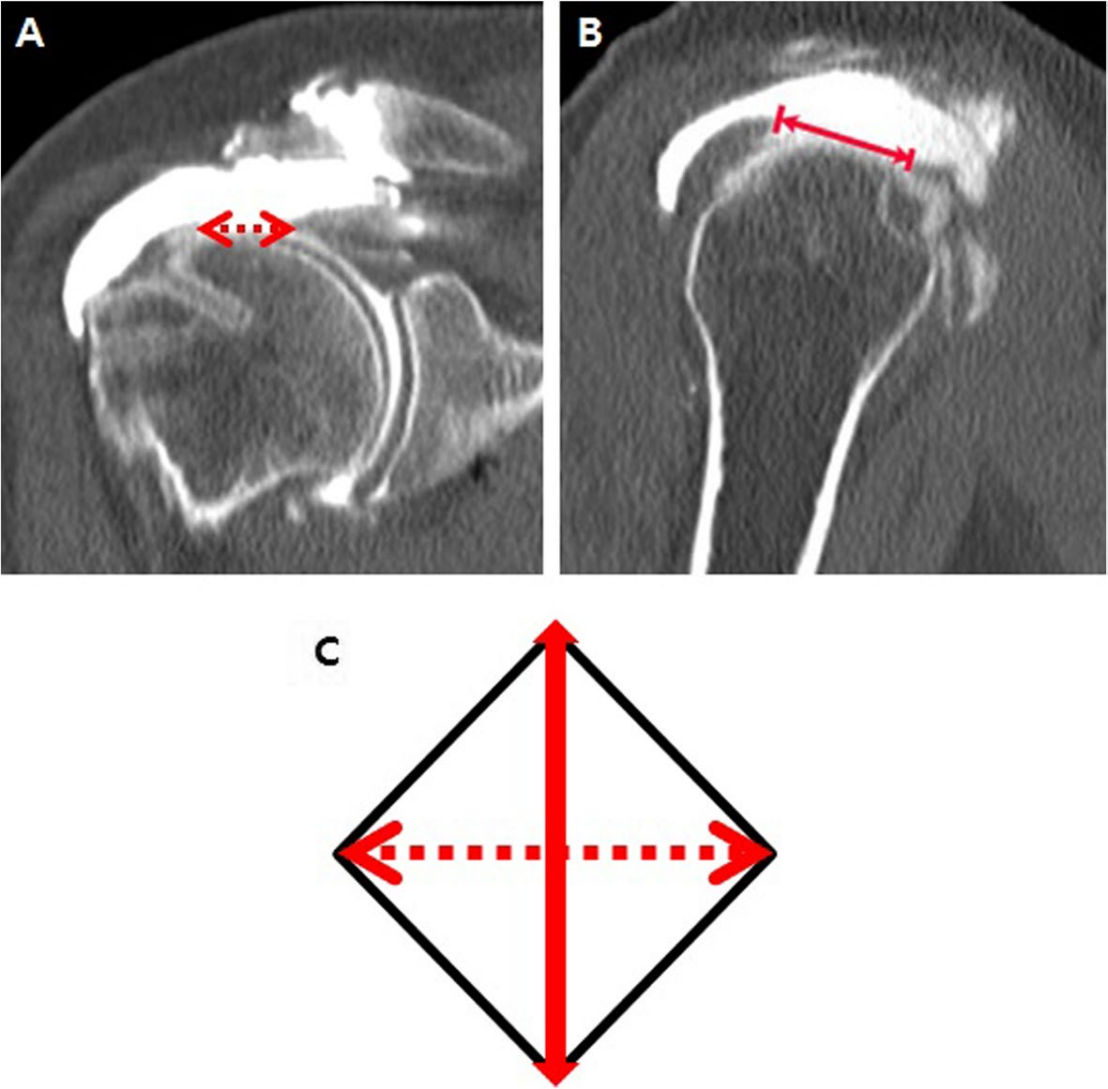
The maximum mediolateral (**a**) and anteroposterior (**b**) diameters of contrast leakage in the coronal and sagittal plane of CT arthrography were measured, respectively. **c** The cross-sectional area of the retorn site was calculated as 1/2 the product of the two diameters, such as the area of the rhombus

The retear size was defined as the cross-sectional area of the retorn site. The maximum anteroposterior (AP) and mediolateral (ML) diameters of the contrast leakage in the sagittal and coronal planes of CTA were measured, respectively. The cross-sectional area of the retorn site was calculated by multiplying half of the AP and ML diameters, such as the area of the rhombus (Fig. 2). All the radiologic parameters were measured using πviewSTAR PACS (INFINITT Co., Seoul, Korea) by two observers in a random manner, and the average values of the two observers’ measurements were used in the data analysis.

### Statistical analysis

The interclass correlation coefficients (ICCs) were calculated to evaluate the interobserver reliability of the retear size and retear type, as assessed by the two observers. One participated in the surgery and the other did not. Correlation analyses were performed to analyze the factors affecting patient satisfaction with surgery. Continuous data, including age, pain duration, ROM, VAS pain level, and ASES score were analyzed using the Mann–Whitney U test, whereas categorical data, including sex, dominant arm, operative history, occupation, worker’s compensation, trauma history, diabetes mellitus, and operative technique were analyzed using the chi-square test or Fisher’s exact test. Independent factors divided into more than three categories were analyzed using the linear-by-linear association method. Post-hoc power analysis was performed to evaluate the validity of the sample size. SPSS version 18.0 (IBM Corp., Armonk, NY, USA) was used for statistical analysis, and a *p* value < 0.05 was considered statistically significant.

## Results

Overall, 39 of 50 patients were classified into the satisfactory group and 11 others into the dissatisfactory group by self-judgment. The mean ages of the satisfactory and dissatisfactory groups were 62.3 years (range, 43–74 years) and 63.4 years (range, 51–72 years), respectively. There was no statistically significant difference in the mean age between the two groups (*P* = 0.679). There were also no differences in the sex, occupation, dominant hand, duration of pain, presence of diabetes mellitus, trauma history, history of ipsilateral shoulder surgery, repair technique, worker’s compensation and duration of follow-up between the two groups. However, the postoperative ASES and VAS pain scores were significantly different (Table 2).

**Table 2:** Correlation between patient factors and satisfaction

			**Satisfactory group (*N* = 39)**	**Unsatisfactory group (*N* = 11)**	***p* value**
**Age**			62.3 (43 ~ 74)	63.4 (51 ~ 72)	0.670^*^
**Preoperative pain duration (months)**			15.1 (1 ~ 60)	11 (1 ~ 24)	0.620^*^
**Range of motion(°)**	**Preop**	**FF**	153.3 (70 ~ 180)	145.9 (30 ~ 180)	0.747^*^
		**ER**	61.6 (30 ~ 80)	61.8 (30 ~ 80)	0.837^*^
	**Postop**	**FF**	155.4 (130 ~ 180)	143.3 (30 ~ 180)	0.672^*^
		**ER**	68.5 (30 ~ 80)	63.6 (50 ~ 80)	0.098^*^
**VAS pain score**	**Preop**		6 (4 ~ 8)	6.6 (5 ~ 10)	0.247^*^
	**Postop**		1.7 (0 ~ 4)	3.9 (2 ~ 6)	< 0.01^*^
**ASES score**	**Preop**		54.9 (42 ~ 71)	55 (35 ~ 68)	0.840^*^
	**Postop**		84.9 (82 ~ 95)	64.5 (62 ~ 71)	< 0.01^*^
**Sex**	**Male**		17	5	0.958^†^
	**Female**		22	6	
**Surgery on dominant side**			26	9	0.193^†^
**Operative history**			1	0	0.703^†^
**Labor- intensive job**			11	3	0.499^†^
**Worker’s compensation**			8	2	0.283^†^
**Trauma history**			2	0	0.601^†^
**Diabetes**			2	1	0.947^†^
**Operative techniques**	**Single row**		20	8	0.601^†^
	**Double row/ suture bridge**		19	3	

^*^Mann–Whitney U test

^†^Chi-square test or Fisher’s exact test

The interobserver reliability of the retear size measured by CTA was judged to be excellent, as the ICCs of the AP and ML diameters of the retorn area were 0.852 and 0.835, respectively. The retear type also showed excellent reliability, with an ICC of 0.764. The retear type and ML diameter of the retorn site showed no significant differences between the two groups. However, the AP diameter and retear size showed statistically significant differences (Table 3). In the post-hoc power analysis to determine the difference between in the retear size the two groups of this sample size, the power was 0.8.

**Table 3:** Correlation between CT arthrography findings and satisfaction

		**Satisfactory group (*n* = 39)**	**Unsatisfactory group (*n* = 11)**	***p* value**
**Anteroposterior diameter (mm)**		5.83 (2 ~ 12)	13 (8 ~ 20)	< 0.01^*^
**Mediolateral diameter (mm)**		4.56 (3 ~ 16)	5.12 (2 ~ 18)	0.542^*^
**Retear size (mm**^**2**^**)**		10.48 (3 ~ 42)	20.45 (8 ~ 160)	< 0.01^*^
**Repaired rotator cuff types** **(footprint attachment status)**	**Ib**	17	6	0.601^†^
	**IIb**	15	2	
	**III**	7	3	

^*^Mann–Whitney U test

^†^Linear-by-linear association

## Discussion

Patient factors were compared between satisfied and dissatisfied patients, and only postoperative VAS pain scores and ASES scores showed significant differences. Correlation analysis between CTA findings and patient satisfaction revealed that retear type did not affect patient satisfaction. However, the dissatisfactory group showed significant correlations with the AP diameter of the retorn site and retear size, indicating that they are risk factors. This is likely because CTA can accurately detect retears, leading to a wider spectrum of retears and ultimately affecting patient satisfaction.

Various methods can be used to assess the integrity of the repaired rotator cuff, including MRI, ultrasonography, and CT [12–14]. However, the reliability and validity of these methods may not be as satisfactory as their pre-operative diagnostic capabilities [15]. Moreover, there is no consensus on which morphological features of the repaired rotator cuff should be considered for repair integrity. Previous studies have typically divided repair integrity into two categories: intact and retorn, or used the Sugaya classification, which has limitations with respect to moderate interobserver reliability and the ability to assess the healing status of the rotator cuff footprint without arthrography [1, 16, 17]. In this study, CTA was used to evaluate repair integrity due to its high sensitivity (99%) and specificity (100%) for detecting supraspinatus tears, and ability to assess the healing status of the footprint [12]. Repair integrity was evaluated based on retear location, shape, and size rather than simply dividing it into intact and retorn. The tear location was categorized into the footprint and medial part because a tear in the footprint can be considered a return to the initial state, while a tear in the medial part is a new tear and expected to have a different clinical outcome. The healing status of the footprint was also expected to impact clinical outcome. However, our study results showed that these factors were not associated with patient satisfaction.

Some studies have reported good clinical outcomes despite retear and suggested that there is no correlation between repair integrity and clinical outcomes [2, 18–20]. However, this contradicts the goal of surgery to anatomically restore the rotator cuff. Indeed, However, this contradicts the goal of surgery to anatomically restore the rotator cuff. McElvany et al. and Russell et al. reported no statistical relationship between repair integrity and clinical outcomes in their studies [1, 15], but other studies have shown that the intact group is superior to the retear group in terms of functional score and muscle strength [10, 21–25]. The relationship between repair integrity and clinical outcome remains ambiguous, and the clinical outcomes of the retear may change over time. As the retraction of a retorn cuff worsens over time, the size of the retear site may also increase, leading to progressively worse clinical outcomes and patient satisfaction.

The study has some limitations that should be considered when interpreting the results. First, CTA was used to evaluate the integrity of the repaired cuff, which may not be able to detect internal changes in the cuff that could be seen on MRI, such as fat infiltration or degenerative changes. The internal quality of the cuff is expected to have an impact on clinical outcomes and satisfaction, similar to signal changes seen in the rotator cuff on MRI in patients with shoulder impingement syndrome. Second, patient satisfaction was used to classify the patient groups, which may be somewhat arbitrary compared to classification based on clinical scores. In addition, patients were not given the option of a middle ground, which may have affected their choices. This could have led to more significant results if a middle ground had been allowed and those patients had been excluded from the statistical analysis. Third, the sample size was small, which may reduce the reliability of the classification. A second-look operation could provide stronger reliability, and further research in this area is needed.

Based on the investigation of retear type and size evaluated by CTA on patient satisfaction, we concluded that although there was no significant relationship between retear types, a larger AP diameter and retear size were associated with a higher likelihood of patient dissatisfaction. Furthermore, patient satisfaction was found to be correlated with postoperative VAS pain scale and ASES score.

## Data Availability

The datasets used and/or analyzed during the current study are available from the corresponding author on reasonable request.

## References

[1] McElvany MD, McGoldrick E, Gee AO, Neradilek MB, Matsen FA (2015). Rotator cuff repair: published evidence on factors associated with repair integrity and clinical outcome. Am J Sports Med.

[2] Oh JH, Kim SH, Ji HM, Jo KH, Bin SW, Gong HS (2008). Prognostic factors affecting anatomic outcome of rotator cuff repair and correlation with functional outcome. Arthroscopy.

[3] Jost B, Zumstein M, Pfirrmann CW, Gerber C (2006). Long-term outcome after structural failure of rotator cuff repairs. J Bone Joint Surg Am.

[4] Paxton ES, Teefey SA, Dahiya N, Keener JD, Yamaguchi K, Galatz LM (2013). Clinical and radiographic outcomes of failed repairs of large or massive rotator cuff tears: minimum ten-year follow-up. J Bone Joint Surg Am.

[5] Hackl M, Flury M, Kolling C, Nebelung W, Krauss CA, Kraemer NA, Heuberer PR, Laky B, Wellmann M, Pastor MF (2022). Results of arthroscopic revision rotator cuff repair for failed open or arthroscopic repair: a prospective multicenter study on 100 cases. Am J Sports Med.

[6] Willinger L, Lacheta L, Beitzel K, Buchmann S, Woertler K, Imhoff AB, Scheiderer B (2018). Clinical outcomes, tendon integrity, and shoulder strength after revision rotator cuff reconstruction: a minimum 2 years’ follow-up. Am J Sports Med.

[7] Tashjian R (2014). Turning failure into success: not always when it comes to the rotator cuff: Commentary on articles by Surena Namdari, MD, MSc, et al., "Factors affecting outcome after structural failure of repaired rotator cuff tears," and H. Mike Kim, MD, et al., "Factors affecting satisfaction and shoulder function in patients with a recurrent rotator cuff tear". J Bone Joint Surg Am.

[8] Yang J, Robbins M, Reilly J, Maerz T, Anderson K (2017). The clinical effect of a rotator cuff retear: a meta-analysis of arthroscopic single-row and double-row repairs. Am J Sports Med.

[9] Kim SC, Shim SB, Kim WJ, Yoo JC (2022). Preoperative rotator cuff tendon integrity, tear size, and muscle atrophy and fatty infiltration are associated with structural outcomes of arthroscopic revision rotator cuff repair. Knee Surg Sports Traumatol Arthrosc.

[10] Kim HM, Caldwell JM, Buza JA, Fink LA, Ahmad CS, Bigliani LU, Levine WN (2014). Factors affecting satisfaction and shoulder function in patients with a recurrent rotator cuff tear. J Bone Joint Surg Am.

[11] Namdari S, Donegan RP, Chamberlain AM, Galatz LM, Yamaguchi K, Keener JD (2014). Factors affecting outcome after structural failure of repaired rotator cuff tears. J Bone Joint Surg Am.

[12] Charousset C, Bellaiche L, Duranthon LD, Grimberg J (2005). Accuracy of CT arthrography in the assessment of tears of the rotator cuff. J Bone Joint Surg Br.

[13] Prickett WD, Teefey SA, Galatz LM, Calfee RP, Middleton WD, Yamaguchi K (2003). Accuracy of ultrasound imaging of the rotator cuff in shoulders that are painful postoperatively. J Bone Joint Surg Am.

[14] Teefey SA, Rubin DA, Middleton WD, Hildebolt CF, Leibold RA, Yamaguchi K (2004). Detection and quantification of rotator cuff tears. Comparison of ultrasonographic, magnetic resonance imaging, and arthroscopic findings in seventy-one consecutive cases. J Bone Joint Surg Am.

[15] Russell RD, Knight JR, Mulligan E, Khazzam MS (2014). Structural integrity after rotator cuff repair does not correlate with patient function and pain: a meta-analysis. J Bone Joint Surg Am.

[16] Sugaya H, Maeda K, Matsuki K, Moriishi J (2005). Functional and structural outcome after arthroscopic full-thickness rotator cuff repair: single-row versus dual-row fixation. Arthroscopy.

[17] Khazzam M, Kuhn JE, Mulligan E, Abboud JA, Baumgarten KM, Brophy RH, Jones GL, Miller B, Smith M, Wright RW (2012). Magnetic resonance imaging identification of rotator cuff retears after repair: interobserver and intraobserver agreement. Am J Sports Med.

[18] Choi CH, Kim SK, Cho MR, Baek SH, Lee JK, Kim SS, Park CM (2012). Functional outcomes and structural integrity after double-pulley suture bridge rotator cuff repair using serial ultrasonographic examination. J Shoulder Elbow Surg.

[19] Kim KC, Shin HD, Cha SM, Kim JH (2013). Repair integrity and functional outcomes for arthroscopic margin convergence of rotator cuff tears. J Bone Joint Surg Am.

[20] Kim KC, Shin HD, Lee WY (2012). Repair integrity and functional outcomes after arthroscopic suture-bridge rotator cuff repair. J Bone Joint Surg Am.

[21] Slabaugh MA, Nho SJ, Grumet RC, Wilson JB, Seroyer ST, Frank RM, Romeo AA, Provencher MT, Verma NN (2010). Does the literature confirm superior clinical results in radiographically healed rotator cuffs after rotator cuff repair?. Arthroscopy.

[22] Cole BJ, McCarty LP, Kang RW, Alford W, Lewis PB, Hayden JK (2007). Arthroscopic rotator cuff repair: prospective functional outcome and repair integrity at minimum 2-year follow-up. J Shoulder Elbow Surg.

[23] Huijsmans PE, Pritchard MP, Berghs BM, van Rooyen KS, Wallace AL, de Beer JF (2007). Arthroscopic rotator cuff repair with double-row fixation. J Bone Joint Surg Am.

[24] Liem D, Lichtenberg S, Magosch P, Habermeyer P (2007). Magnetic resonance imaging of arthroscopic supraspinatus tendon repair. J Bone Joint Surg Am.

[25] Jost B, Pfirrmann CW, Gerber C, Switzerland Z (2000). Clinical outcome after structural failure of rotator cuff repairs. J Bone Joint Surg Am.

